# Portal vein stent to save the day

**DOI:** 10.1186/s42155-025-00586-3

**Published:** 2025-08-29

**Authors:** Panagiotis Grigoropoulos, Vasilina Tsemelidi, Orfeas Varvarelis, Antonios Vezakis, Achilles Chatziioannou, Miltiadis Krokidis

**Affiliations:** 1 1st Department of Radiology, School of Medicine, National and Kapodistrian University of Athens, Areteion Hospital, 76 Vas. Sophias Ave, Athens, 11528 Greece; 2 2nd Department of Surgery, School of Medicine, National and Kapodistrian University of Athens, Areteion Hospital, Athens, Greece

**Keywords:** Portal vein stenosis/occlusion, Portal venous stent, Hepatic failure, Whipple's procedure

## Abstract

**Background:**

Post-operative portal vein stenosis/occlusion is a relatively common and life-threatening condition. Clinical outcomes depend on the early diagnosis and the appropriate management.

**Case presentation:**

A 78-year-old female developed portal vein high grade stenosis after resection of an ampullary cholangiocarcinoma. The patient presented with acute liver failure and impaired coagulation function. Imaging confirmed transient ischemic changes of the hepatic parenchyma. Salvage attempt with a portal venous stent was decided to prevent irreversible liver damage. The procedure was successful with a satisfactory clinical outcome.

**Conclusions:**

The paper offers a summary of the clinical benefit from percutaneous portal venous stent placement in case of post operative high grade portal vein stenosis in a patient with acute liver failure.

## Background

Clinical focus on long-term complications following Whipple’s procedure has recently increased. Several studies highlight portal vein (PV) stenosis as a significant long-term complication, particularly in patients who have undergone portal vein resection or received chemoradiotherapy, with an incidence of up to 19.6% [1]. Surgical management of PV stenosis can lead to severe bleeding due to adhesions and rich collateral vessels, whereas stent placement is a minimally invasive, highly effective, and safe technique for managing symptomatic PV stenosis from a long-term perspective [2]. You et al. reported satisfactory outcomes in 15 patients treated with portal venous stenting over 18 years, addressing symptoms such as gastrointestinal bleeding or ascites caused by portal hypertension associated with PV stenosis/occlusion, typically after a median of 19 months post-surgery [2]. Similar approaches are noted in other studies where stenting was performed to alleviate portal hypertension symptoms [3, 4]. Khan et al. in 23 patients that underwent percutaneous transhepatic portal vein stent placement post liver transplantation or hepato-pancreato-biliary surgery reported satisfactory outcomes with 100% technical success and 80% clinical success [4]. However, two procedure-related hemorrhages and two early stent thromboses occurred [4]. Kinota et al. also demonstrated significant improvement in hepatic reserve scores following portal vein stenting in 25 patients with severe impaired hepatic function post-Whipple’s procedure, particularly in those with severe hypoalbuminemia [5]. In the same series, 9 late stent failures occurred during a median follow-up period of 543 days [5]. On the other hand, surgical approach is linked with significant morbidity and mortality as confirmed by a recently published systematic review [6]. Nevertheless, most cases, typically involve patients with normal coagulation parameters, unlike the challenging scenario presented in our case.

### Case presentation

**Fig. 1:**
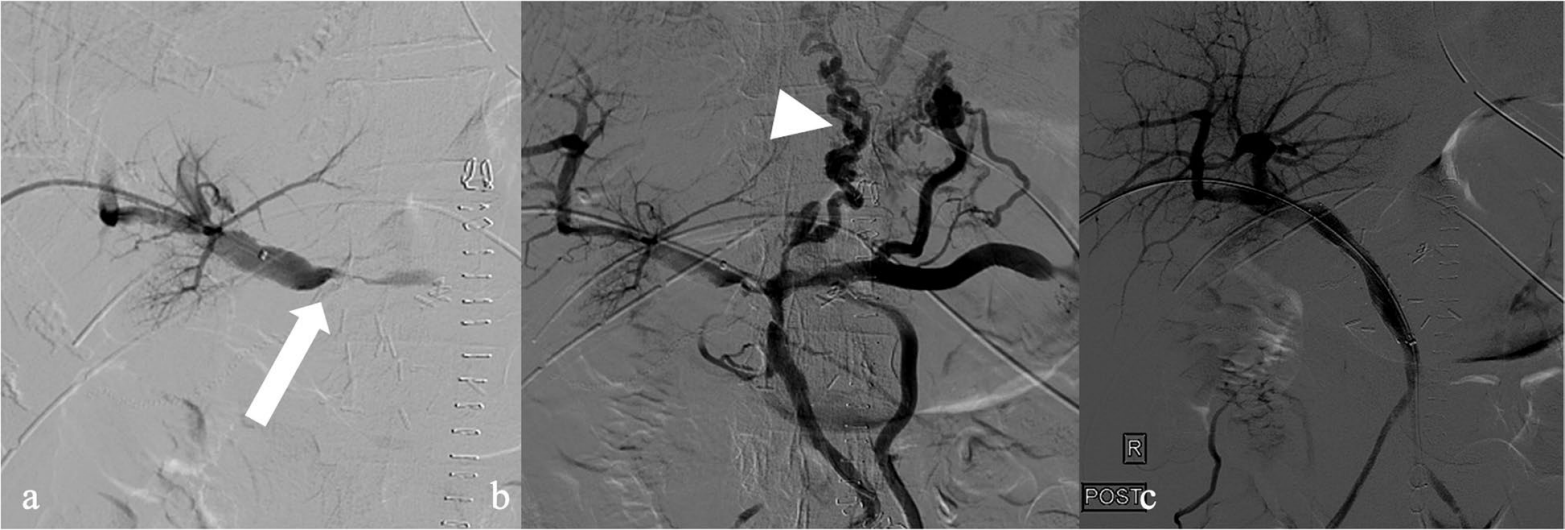
a) Portography from a right-side access confirming the tight stenosis of the portal vein at the level of the portal bifurcation (arrow). b) Recanalization of the stenotic area and contrast injection confirmed extensive gastric varices (arrow head). c) Post stent deployment satisfactory flow with lack of perfusion in the varices.

**Fig. 2:**
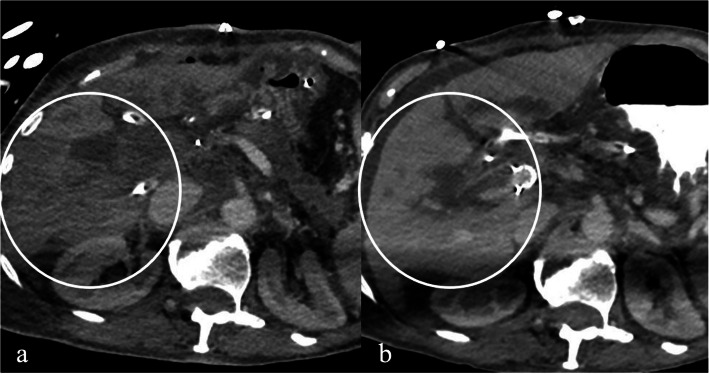
a) CT scan prior to portal vein stent confirming portal vein occlusion and transient hepatic attenuation differences in the early portal phase (circle). b) Liver perfusion significantly improved with no further attenuation differences (circle) and patent portal vein

We report the case of a 78-year-old female with a known cholangiocarcinoma of the ampulla of Vater, presenting to our center for surgical treatment. Her past medical history included type 2 diabetes mellitus, hypertension, dyslipidemia, and paroxysmal atrial fibrillation. She had received six cycles of neoadjuvant chemotherapy over three months and undergone multiple endoscopic retrograde cholangiopancreatographies (ERCPs) due to cholangitis prior to her latest admission. The patient underwent Whipple’s procedure without vascular reconstruction, with suturing of the pancreatic stump. Post-operatively, she was transferred to the intensive care unit for monitoring. On post-operative day (POD) 1, triplex sonographic evaluation of the splenoportal axis indicated low flow velocity. A contrast CT scan on POD 2 confirmed portal vein occlusion and transient hepatic attenuation differences (THAD) in the arterial/early portal phase, suggesting liver parenchyma failure. Over the next few days, the patient developed portal hypertension, manifesting as ascites, encephalopathy, low platelet count (21,000/ μL), elevated hepatic enzymes (Alanine transaminase (ALT): 230 IU/L, aspartate transaminase (AST): 331 IU/L, alkaline phosphatase (ALP): 670 IU/L, and gamma-glutamyltransferase (GGT): 873 IU/L), and increased bilirubin (4.2 mg/dl). On POD 5, due to imminent liver failure, the multidisciplinary team decided to place a portal vein stent. The patient’s coagulation status was optimized with two bags of fresh frozen plasma and two bags of platelets, as her prothrombin time international ratio was 3.4 and platelet count was 21,000/µL. An ultrasound-guided puncture of the right-side portal vein was performed with a 21G needle system (AccuStick™, Boston Scientific, USA), and a 5-Fr sheath was inserted. Portography confirmed tight stenosis of the main portal vein branch with jejunal varices, retrograde flow in the splenic vein, and gastric varices (Figs. 1a and 1b). Recanalization to the superior mesenteric vein was achieved, followed by deployment of a 14 mm × 6 cm self-expandable metallic stent (Sinus-XL 6F, Optimed, Germany) in the stenotic region. Post-dilation with an 8 mm balloon catheter was performed. Post-stent portography confirmed no residual stenosis and adequate flow. The sheath track was embolized with an 8 mm × 5 cm coil, with no bleeding observed. The patient remained hemodynamically stable and was extubated on POD 8. She was transferred to the general ward, and her liver function normalized (ALT: 47 IU/L, AST: 38 IU/L, ALP: 100 IU/L, GGT: 31 IU/L). A follow-up CT scan confirmed improved liver perfusion, and she was discharged two weeks later. While in the hospital the patient received treatment dose of low molecular weight heparin and when discharged received anticoagulation with 15 mg daily of Rivaroxaban. At the three-month follow-up, the patient was in good condition with normal liver function. Follow-up with CT scan every three months was arranged. (Fig 2)

## Conclusions

This case highlights the successful management of acute liver failure due to portal vein occlusion following Whipple’s procedure using emergency portal vein stenting. Unlike previous reports where stenting was performed for chronic symptoms of portal hypertension, this intervention was considered necessary due to the rapid patient deterioration and the acute liver failure, compounded by deranged coagulation status. Despite these challenges optimization of coagulation parameters and appropriate stenting technique enabled safe and effective stent placement. Kinota et al. noted improved hepatic reserve post-stenting, but their patients had normal coagulation profiles [5]. In case of deranged coagulation status like in our case the initial measure consists of supplying coagulation products to the patient (platelets and fresh frozen plasma) and trying to obtain access with the minimal risk (small size needle and single ultrasound guided puncture). To avoid any track bleeding, embolization with coils, glue or sponge needs to be performed like in our case. Our case demonstrates that stenting is feasible even in acute settings with compromised coagulation, provided appropriate optimization is performed. One potential complication following portal venous stent placement is stent occlusion especially if residual stenosis after stent placement is greater than 30%, so complete release of stenosis during the procedure is crucial [6]. Measures to prevent occlusion mainly consist in oversizing a bit the stent (at least 2 mm), obtaining satisfactory stent expansion which in the case of self-expandable stents may be achieved with the use of balloon dilatation post deployment and administering anticoagulation with either low molecular weight heparin (150 IU/kg/day) or Direct Oral Anticoagulants (DOACs) for a minimum of 3 months [7].

Even though portal vein stenting is not a novel procedure, we considered communicating this experience because we had to deal with challenging, rapidly deteriorating case that was resolved with a minimal invasive percutaneous solution.

## Data Availability

Not applicable.

## Abbreviations

ERCP: Endoscopic Retrograde Cholangiopancreatography
POD: Post-Operative Day
CT: Computed Tomography
THAD: Transient Hepatic Attenuation Differences
PV: Portal Vein
Fr: French (unit for catheter/sheath size, e.g., 5-Fr sheath)
mm: Millimeters
cm: Centimeters
μL: Microliter
ALT: Alanine transaminase
AST: Aspartate transaminase
ALP: Alkaline phosphatase
GGT: Gamma-glutamyltransferase
DOACs: Direct Oral Anticoagulants
